# SARS-CoV-2 can recruit a heme metabolite to evade antibody immunity

**DOI:** 10.1126/sciadv.abg7607

**Published:** 2021-05-28

**Authors:** Annachiara Rosa, Valerie E. Pye, Carl Graham, Luke Muir, Jeffrey Seow, Kevin W. Ng, Nicola J. Cook, Chloe Rees-Spear, Eleanor Parker, Mariana Silva dos Santos, Carolina Rosadas, Alberto Susana, Hefin Rhys, Andrea Nans, Laura Masino, Chloe Roustan, Evangelos Christodoulou, Rachel Ulferts, Antoni G. Wrobel, Charlotte-Eve Short, Michael Fertleman, Rogier W. Sanders, Judith Heaney, Moira Spyer, Svend Kjær, Andy Riddell, Michael H. Malim, Rupert Beale, James I. MacRae, Graham P. Taylor, Eleni Nastouli, Marit J. van Gils, Peter B. Rosenthal, Massimo Pizzato, Myra O. McClure, Richard S. Tedder, George Kassiotis, Laura E. McCoy, Katie J. Doores, Peter Cherepanov

**Affiliations:** 1 Chromatin Structure and Mobile DNA Laboratory, The Francis Crick Institute, London, UK.; 2 Department of Infectious Diseases, School of Immunology and Microbial Sciences, King's College London, London, UK.; 3 Institute of Immunity and Transplantation, Division of Infection and Immunity, University College London, London, UK.; 4 Retroviral Immunology Laboratory, The Francis Crick Institute, London, UK.; 5 Department of Infectious Disease, St. Mary’s Campus, Imperial College London, London, UK.; 6 Metabolomics Science Technology Platform, The Francis Crick Institute, London, UK.; 7 Department of Cellular, Computational and Integrative Biology, University of Trento, Trento, Italy.; 8 Flow Cytometry Science and Technology Platform, The Francis Crick Institute, London, UK.; 9 Structural Biology Science Technology Platform, The Francis Crick Institute, London, UK.; 10 Cell Biology of Infection Laboratory, The Francis Crick Institute, London, UK.; 11 Structural Biology of Disease Processes Laboratory, The Francis Crick Institute, London, UK.; 12 Cutrale Perioperative and Ageing Group, Imperial College London, London, UK.; 13 Department of Medical Microbiology, Amsterdam UMC, University of Amsterdam, Amsterdam Institute for Infection and Immunity, Amsterdam, Netherlands.; 14 Weill Medical College of Cornell University, New York, NY, USA.; 15 Advanced Pathogen Diagnostic Unit, University College London Hospitals NHS Foundation Trust, London, UK.; 16 Crick COVID-19 Consortium, The Francis Crick Institute, London, UK.; 17 Department of Infection, Immunity and Inflammation, UCL Great Ormond Street Institute of Child Health.; 18 Structural Biology of Cells and Viruses Laboratory, The Francis Crick Institute, London, UK.

## Abstract

The coronaviral spike is the dominant viral antigen and the target of neutralizing antibodies. We show that SARS-CoV-2 spike binds biliverdin and bilirubin, the tetrapyrrole products of heme metabolism, with nanomolar affinity. Using cryo–electron microscopy and x-ray crystallography, we mapped the tetrapyrrole interaction pocket to a deep cleft on the spike N-terminal domain (NTD). At physiological concentrations, biliverdin significantly dampened the reactivity of SARS-CoV-2 spike with immune sera and inhibited a subset of neutralizing antibodies. Access to the tetrapyrrole-sensitive epitope is gated by a flexible loop on the distal face of the NTD. Accompanied by profound conformational changes in the NTD, antibody binding requires relocation of the gating loop, which folds into the cleft vacated by the metabolite. Our results indicate that SARS-CoV-2 spike NTD harbors a dominant epitope, access to which can be controlled by an allosteric mechanism that is regulated through recruitment of a metabolite.

## Teaser

SARS-CoV-2 spike N-terminal domain harbors a potent epitope that can be modulated by binding of natural linear tetrapyrroles.

## INTRODUCTION

Trimeric coronaviral spike glycoproteins form prominent features on viral particles that are responsible for the attachment to a receptor on the host cell and, ultimately, fusion of the viral and cellular membranes (*1*, *2*). Encoded by a single viral gene, the mature spike glycoprotein comprises two subunits, S1 and S2, which mediate binding to the receptor and facilitate fusion, respectively. The recognition of the betacoronavirus SARS-CoV-2 host receptor, the cellular membrane protein angiotensin-converting enzyme 2 (ACE2), maps to the S1 C-terminal domain (referred to as the receptor binding domain, RBD) (*3*–*5*), while the function of the N-terminal domain (NTD) remains enigmatic. The immune properties of the spike glycoprotein underpin ongoing SARS-CoV-2 vaccine development efforts (*6*). Both S1 domains can be targeted by potent neutralizing antibodies that arise in infected individuals. Most of the characterized neutralizing antibodies bind the RBD, while minimal structural information exists about neutralizing epitopes on the NTD (*7*–*11*). Mutations within the SARS-CoV-2 spike NTD are associated with viral escape from antibody immunity (*12*–*14*) and have been observed in circulating viral strains (*15*, *16*).

Here we show that the NTD of SARS-CoV-2 spike binds biliverdin with high affinity. The recruitment of the tetrapyrrole leads to stabilization of the NTD structure and makes the viral envelope glycoprotein refractory to neutralization by a subset of human antibodies. Using cryo–electron microscopy (cryo-EM) and x-ray crystallography, we dissect the mechanism of the competition between the metabolite and antibodies for binding to the spike.

## RESULTS

### SARS-CoV-2 and SARS-CoV-1 spike NTDs bind biliverdin and bilirubin

In the course of our activities to support the development of serology for SARS-CoV-2, we produced a range of recombinant coronaviral spike antigens by expression in human cell lines (fig. S1A). Unexpectedly, preparations of SARS-CoV-2 trimeric spike and S1 carried a distinctive green hue, with prominent peaks at ~390 and 670 nm in their light absorbance spectra (fig. S1, A and B). These unusual features were also evident in the spectrum of S1 from SARS-CoV-1, another member of the sarbecovirus subgenus, but not those from the seasonal human coronaviruses NL63 and OC43 (fig. S1, B and C). The property was confined within the spike NTD and absent in isolated RBD (fig. S1B). The spectra of the SARS-CoV spike constructs were consistent with biliverdin (fig. S1B), a product of heme metabolism responsible for the coloration of bruises and green jaundice. We isolated the pigment from denatured SARS-CoV-2 S1 and confirmed the presence of biliverdin IXα by mass spectrometry (MS) (fig. S2).

Heme is an essential prosthetic group in many proteins involved in processes requiring oxygen and/or electron transfer, such as cellular respiration (e.g., mitochondrial cytochrome c oxidase), oxygenation (P450 cytochromes), nitric oxide, and peroxide metabolism (*17*). Because free heme is highly toxic, its cellular levels are tightly regulated through constitutive synthesis and catabolism. Biliverdin is produced at the first step of endogenous heme detoxification through the action of oxygenases and is then reduced to bilirubin, which is the final product of tetrapyrrole catabolism in humans. We measured tetrapyrrole binding to immobilized SARS-CoV-2 S1 using surface plasmon resonance (SPR) and estimated the dissociation constant (*K*_d_) for the interaction with biliverdin and bilirubin at 9.8 ± 1.3 nM and 720 ± 250 nM, respectively (fig. S3, A and B, and table S1). S1 bound heme considerably more weakly, with a *K*_d_ of 7.0 ± 1.2 μM, while no interaction was observed with protoporphyrin IX, the intact macrocyclic tetrapyrrole of heme (fig. S3, C and D, and table S1).

### Structural basis for biliverdin recruitment by SARS-CoV-2 spike

**Fig. 1:**
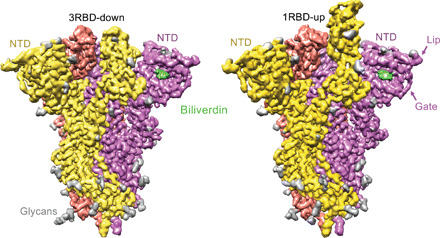
Cryo-EM structures of the SARS-CoV-2 spike-biliverdin complex. Three-dimensional reconstructions of trimeric SARS-CoV-2 spike ectodomain in 3RBD-down (left) and 1RBD-up (right) conformations determined under saturation with biliverdin. Spike protomers are color-coded. Biliverdin and glycans are shown in green and gray, respectively.

**Fig. 2:**
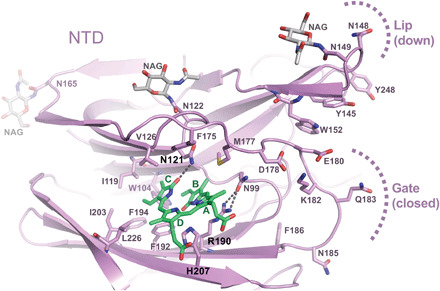
Crystal structure of isolated SARS-CoV-2 spike NTD bound to biliverdin. Details of the biliverdin binding pocket in the crystal structure refined at 1.8-Å resolution. SARS-CoV-2 NTD is shown as cartoons with selected amino acid residues and biliverdin in sticks. Carbon atoms of the protein chain, sugars (N-acetylglucosamine, NAG), and biliverdin are in purple, gray, and green, respectively; the remaining atoms are colored as follows: oxygen, red; nitrogen, blue; and sulfur, yellow. Dark gray dashes are hydrogen bonds.

Next, we imaged single particles of the trimeric SARS-CoV-2 spike ectodomain (*3*, *18*) in the presence of excess biliverdin using cryo-EM. Image processing resulted in the three-dimensional (3D) reconstruction of closed (3RBD-down) and partially open (1RBD-up conformation) states of the spike at 3.35- and 3.50-Å resolution, respectively (Fig. 1, fig. S4, and table S2). Close inspection of the cryo-EM maps revealed features interpretable as a biliverdin molecule buried within a deep cleft on one side of each of the NTD domains (Fig. 1 and fig. S5A). To define the structural basis for the interaction more precisely, we cocrystallized the isolated NTD with biliverdin and determined the structure at 1.8-Å resolution (Fig. 2, fig. S5B, and table S3). The metabolite fits snugly into the cleft, with the pyrrole rings B and C buried inside and propionate groups appended to rings A and D projecting toward the outside. The pocket is lined by hydrophobic residues (Ile^101^, Trp^104^, Ile^119^, Val^126^, Met^177^, Phe^192^, Phe^194^, Ile^203^, and Leu^226^), which form van der Waals interactions with the ligand. Biliverdin packs against His^207^, which projects its Nε2 atom toward pyrrolic amines, approaching three of them at ~3.6 Å. Pyrroles A and B are involved in a π-π stacking with side chain of Arg^190^, which is stabilized by hydrogen bonding with Asn^99^. The binding of biliverdin largely buries the side chain of Asn^121^, which makes a hydrogen bond with the lactam group of pyrrole D. In agreement with the extensive interactions observed in the crystal structure, the melting point of isolated NTD increased by over 8°C in the presence of biliverdin (fig. S3K). Unidentified entities at the tetrapyrrole binding site were observed in published SARS-CoV-2 spike reconstructions (*1*, *4*, *18*–*22*), presumably obtained with partial occupancy by the metabolite; in some cryo-EM maps, the bound biliverdin molecule is resolved remarkably well (fig. S6) (*19*–*22*).

We speculated that the protonation state of His^207^ in the biliverdin binding pocket (Fig. 2), and indeed of the tetrapyrrole itself, may affect the interaction. Concordantly, the *K*_d_ of the S1-biliverdin interaction increased to 250 ± 100 μM at pH 5.0 (fig. S3, E and H, and table S1), and purification under acidic conditions greatly reduced the biliverdin content of recombinant SARS-CoV-2 S1 (fig. S1D). Substitutions of spike residues closely involved in ligand binding (H207A, R190K, and N121Q) diminished pigmentation of purified recombinant protein (fig. S1E). The biliverdin binding affinity of SARS-CoV-2 S1 was reduced by two and three orders of magnitude by the R190K and N121Q amino acid substitutions, respectively (fig. S3, F to H, and table S1). The latter ablated the interaction with bilirubin, confirming that the tetrapyrroles share the binding site on the spike (fig. S3H). The residues corresponding to Asn^121^ and Arg^190^ appear to be strictly conserved across the entire sarbecovirus subgenus. By contrast, position 207 is variable, usually occupied by a tyrosine residue, as is the case in SARS-CoV-1 (fig. S7). Because SARS-CoV-1 S1 retained nanomolar affinity for biliverdin (*K*_d_ = 19.6 ± 0.8 nM; fig. S3I and table S1), the tetrapyrrole binding may be a common property of sarbecoviral spike glycoproteins.

### Biliverdin binding significantly down-modulates the reactivity of SARS-CoV-2 spike with immune sera

**Fig. 3:**
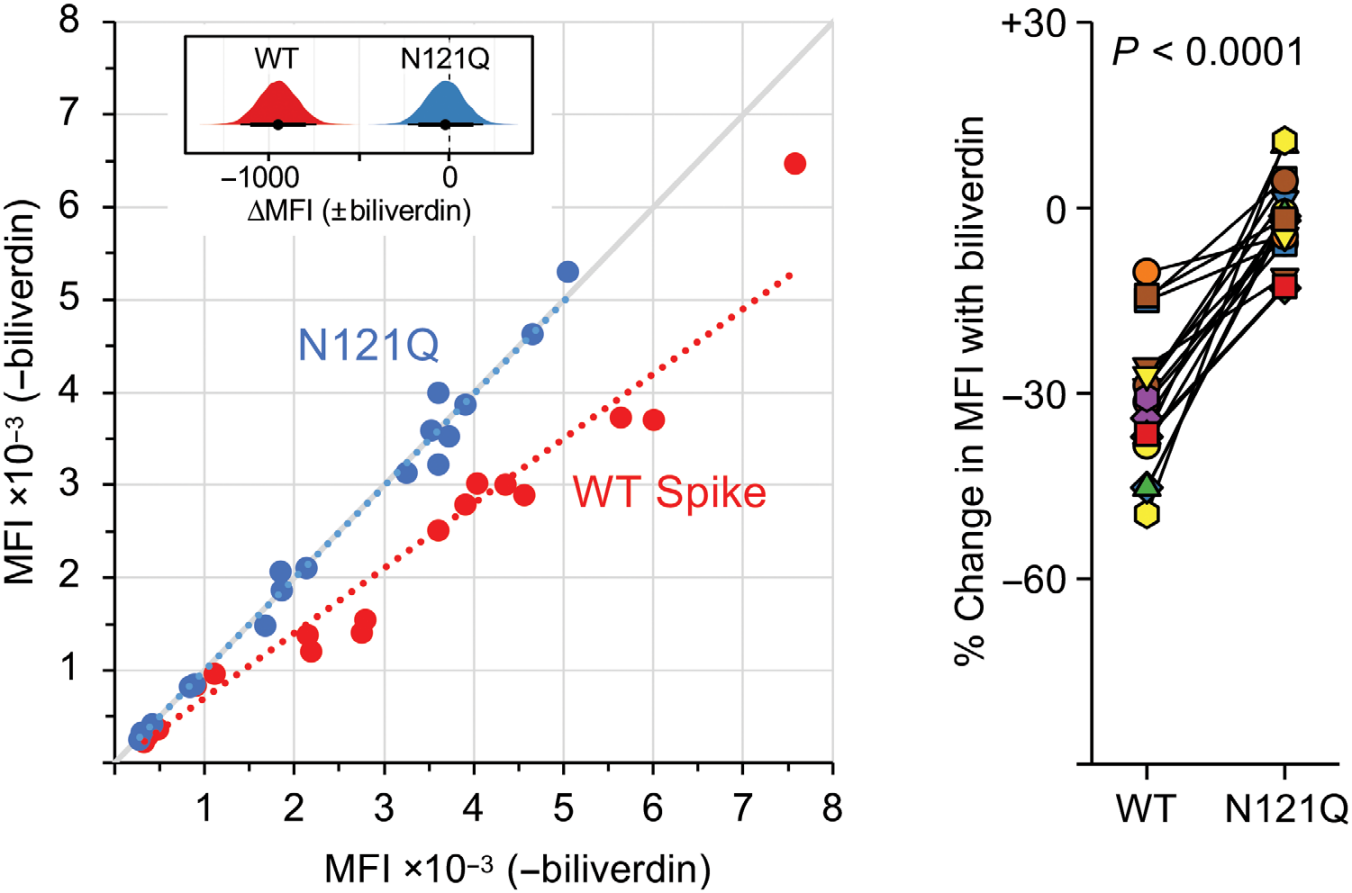
Biliverdin strongly down-modulates the reactivity of SARS-CoV-2 spike with antibodies present in immune sera. (**Left**) Mean fluorescence intensity (MFI) of IgG staining of human embryonic kidney (HEK) 293T cells expressing full-length WT or N121Q SARS-CoV-2 spike by individual patient sera in the absence or the presence of 10 μM biliverdin. Each symbol represents an individual patient (*n* = 17), and colored dotted lines represent the linear regression for each spike variant. The inset shows posterior probability density plots of values for pairwise contrasts (±biliverdin) for the WT and N121Q spikes. Black dots indicate the median of the distribution, and thick and thin line ranges correspond to the 85 and 95% highest density interval, respectively; the dotted vertical line indicates a zero difference. (**Right**) Changes in MFI caused by the addition of 10 μM biliverdin, as percent of staining without biliverdin, for serum for IgG antibodies. Each pair of connected symbols represents an individual patient. The *P* value reported above the plot was calculated using a two-tailed paired Student’s *t* test comparing the effect of biliverdin (percent change in binding) on the WT spike versus the effect of biliverdin on the N121Q spike for each serum sample.

SARS-CoV-2 spikes carrying R190K and N121Q mutations supported infection of Vero-ACE2 and Huh7-ACE2 cells by a pseudotyped lentiviral vector (fig. S8A). Moreover, the infectivity of replication-competent SARS-CoV-2 was not affected by the presence of 100 μM biliverdin (fig. S8B). Thus, the metabolite does not appear to play a critical role in viral entry under standard tissue culture conditions. Because biliverdin binding conceals a deep hydrophobic cleft on the NTD (Fig. 2), we suspected that it may mask or modify the antigenic properties of the viral spike. To test this hypothesis, we measured the reactivity of sera from 17 SARS-CoV-2–infected and convalescent individuals with full-length wild-type (WT) and N121Q SARS-CoV-2 spike using a flow cytometry–based assay (*23*). The addition of 10 μM biliverdin reduced binding of patient immunoglobulin Gs (IgGs) to WT spike, reducing the reactivity of some of the immune sera by as much as 50% (with a median change of −31.4%, ranging from −10.4 to −49.5%) (Fig. 3). By contrast, antibody binding to N121Q SARS-CoV-2 spike was not affected by addition of biliverdin (median change of −2.0%, ranging from +10.8 to −12.9%) (Fig. 3). Binding of IgM and IgA antibodies, which are present at lower titers in these patients (*23*), was more variably, but statistically, not significantly affected (fig. S9, B to D). In a separate experiment, we tested 91 clinical serum samples in an IgG capture enzyme-linked immunosorbent assay (ELISA). SARS-CoV-2–specific antibodies were detected using WT or N121Q S1 conjugated to horseradish peroxidase (HRP). Biliverdin-depleted WT S1 was significantly more reactive than the same protein supplemented with excess metabolite (fig. S10). By contrast, addition of biliverdin did not dampen detection of the SARS-CoV-2 antibodies with N121Q S1 (fig. S10).

### Identification of biliverdin-sensitive monoclonal antibodies to SARS-CoV-2 spike NTD

**Fig. 4:**
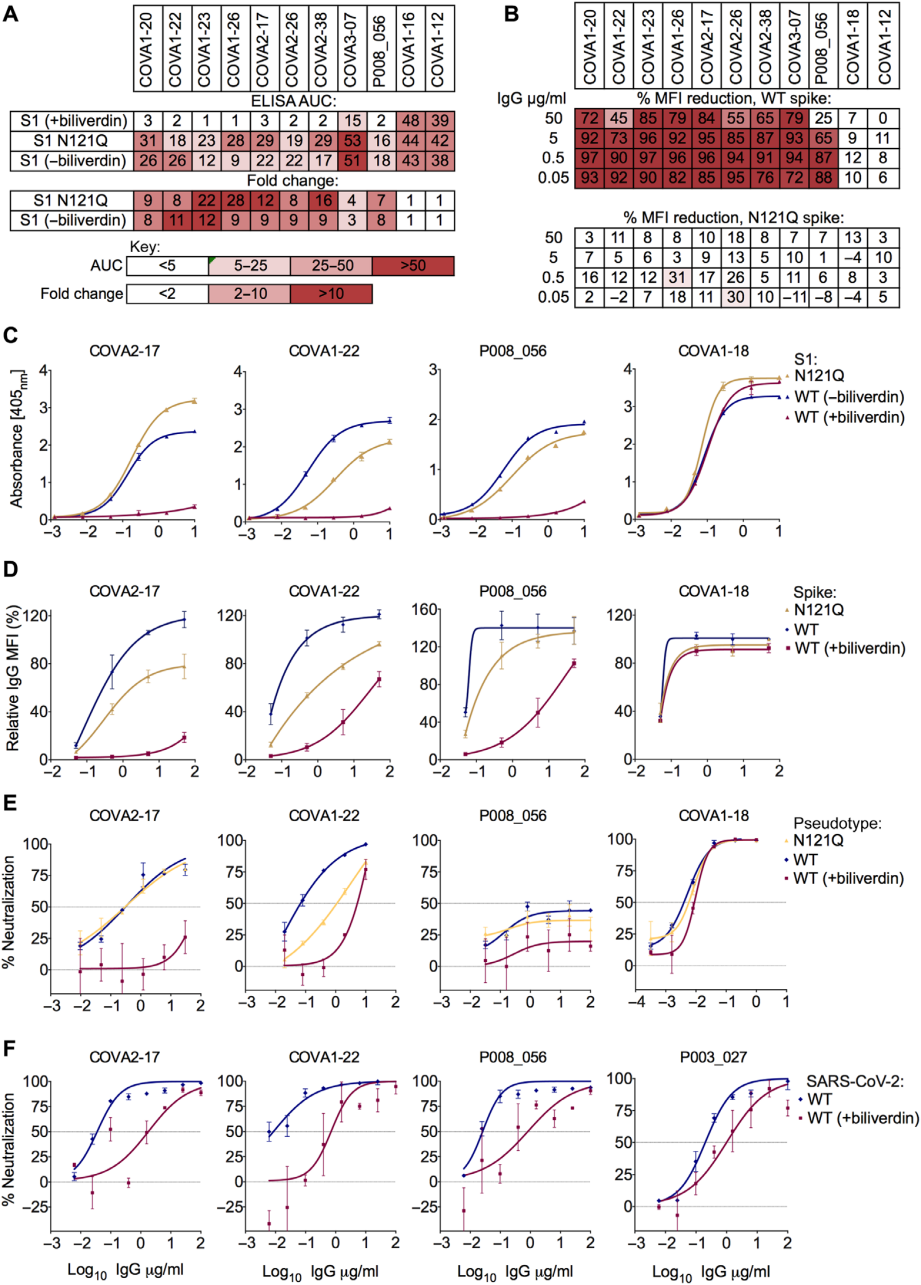
Biliverdin decreases binding to SARS-CoV-2 spike by a group of human monoclonal IgGs. (**A**) Antibodies were titrated sixfold and assayed by direct ELISA for binding to recombinant S1 biliverdin-depleted by purification under acidic conditions (−biliverdin), same protein but supplemented with biliverdin (+biliverdin) or N121Q S1. Area under the curve (AUC) is shown for IgG that were sensitive to biliverdin and two unaffected control IgGs. AUC values are color-coded as per the key; fold change compared to WT protein are reported. (**B**) Biliverdin-sensitive IgGs were titrated 10-fold and incubated with 293T cells expressing full-length WT or N121Q SARS-CoV-2 spike with or without 10 μM biliverdin. Binding was detected using an anti-IgG antibody and reduction in binding in the presence of biliverdin is shown as % MFI reduction and color-coded as a heatmap of the quartile values. (**C**) Enzyme-linked immunosorbent assay (ELISA) titration curves for four neutralizing IgG including the biliverdin-insensitive control COVA1-18. (**D**) Relative MFI dose-dependent curves for four neutralizing IgG including the biliverdin-insensitive control COVA1-18. Relative MFI calculated by normalizing to the MFI of the biliverdin-insensitive COVA1-18 at the highest concentration against spike. (**E**) IgG indicated above each graph were titrated fivefold against SARS-CoV-2 spike pseudotype, in the presence and absence of 10 μM biliverdin, and a version of spike encoding the mutation N121Q. COVA1-18 was used as a biliverdin-insensitive control IgG. (**F**) Neutralization of SARS-CoV-2 (England 02/2020/407073) by IgGs was measured in the absence and presence of 10 μM biliverdin in Vero-E6 cells. P003_027 was used as a biliverdin-insensitive control IgG.

It is remarkable that a small molecule with a footprint of 370 Å^2^, corresponding to less than 0.9% of solvent-exposed surface (per spike monomer; Fig. 1), competes with a considerable fraction of the spike-specific serum antibody population (Fig. 3). These results prompted us to evaluate a panel of human antibodies cloned from B cells of SARS-CoV-2 convalescent individuals. We used 38 IgGs reported in a recent study (*11*), as well as a panel of 15 novel SARS-CoV-2 S1-specific IgGs obtained from individuals with asymptomatic infection or mild/severe disease undergoing characterization in one of our laboratories (*14*). We tested these monoclonal antibodies for binding to recombinant WT and N121Q S1 by ELISA. Nine of 53 (17%) IgGs lost binding to WT, but not to N121Q S1, in the presence of 10 μM biliverdin (Fig. 4, A and C, and fig. S11A). Furthermore, addition of biliverdin strongly suppressed binding of these antibodies to full-length WT but not N121Q SARS-CoV-2 spike expressed on the surface of transfected human embryonic kidney (HEK) 293T cells (Fig. 4, B and D). By contrast, the reactivity of both RBD-specific controls (COVA1-18 and COVA1-12) was not affected by the metabolite (Fig. 4, A to D). As expected, all biliverdin-sensitive antibodies recognize the NTD (*11*, *14*).

Two of the biliverdin-sensitive monoclonal IgGs, COVA1-22 and COVA2-17, were previously reported to efficiently neutralize SARS-CoV-2–pseudotyped retrovirus (*11*). Addition of biliverdin suppressed neutralization of the pseudotype carrying WT but not N121Q spike (Fig. 4E). The mutation had a differential effect on neutralization showing a decrease in potency for COVA1-22 and no effect for P008_056 or COVA2-17. As expected, addition of biliverdin had no effect on neutralization by COVA1-18. Neutralization of replication-competent SARS-CoV-2 by P008_056, COVA1-22, or COVA2-17 was substantially decreased by addition of biliverdin (Fig. 4F). Intriguingly, while P008_056 seemed somewhat less potent in the pseudotype assay (Fig. 4E), it efficiently neutralized live SARS-CoV-2 virus, achieving 50 and >90% inhibition at concentrations of 0.03 and 1.56 μg/ml, respectively, in a biliverdin-sensitive manner (Fig. 4F and fig. S11B).

### Biliverdin inhibits antibody binding to SARS-CoV-2 spike NTD via an allosteric mechanism

**Fig. 5:**
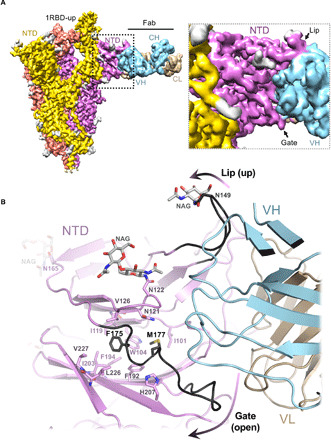
Cryo-EM structure of the spike-Fab complex. (**A**) Reconstruction obtained with multibody refinement in Relion (left) and a zoom on the spike-Fab interface in the structure obtained by consensus refinement (fig. S12D). (**B**) Refined model of the spike-Fab complex shown as cartoon, with selected amino acid side chains in sticks and indicated. Carbon atoms of the gate and lip NTD elements that relocate to allow Fab binding (arrows) are shown in black. Fab heavy (HV) and light (LV) chains are shown in blue and beige, respectively.

To establish the structural basis for SARS-CoV-2 neutralization by a biliverdin-sensitive antibody, we imaged single particles of the viral spike in complex with P008_056 antigen binding fragment (Fab). Cryo-EM image processing resulted in reconstruction of structures with one, two, and three Fab moieties bound per trimeric viral glycoprotein (fig. S11, A and B), and the best map was obtained for the complex containing a single Fab (Fig. 5A and fig. S12, C and D). The reconstruction with a local resolution of ~4 Å at the NTD-Fab interface allowed for tracing of the protein backbone and revealed positions of key amino acid side chains (Fig. 5A and fig. S5C). P008_056 binds the spike at the side of the NTD β-sandwich fold, which undergoes profound conformational rearrangements (movie S1). Access to the epitope is gated by a solvent-exposed loop composed of predominantly hydrophilic residues (“gate,” SARS-CoV-2 spike residues 174 to 188; Fig. 2). To allow P008_056 binding, the loop swings out of the way, with a backbone displacement in the middle of the loop of ~15 Å (Fig. 5B). The gating mechanism is accompanied by insertion of Phe^175^ and Met^177^, which are located in the beginning of the loop, into the hydrophobic pocket vacated by biliverdin (Fig. 5B and fig. S5C). Thus, when bound, the metabolite appears to act as a wedge that restricts gate opening. Antibody binding is additionally complemented by an upward movement of a β-hairpin (“lip,” SARS-CoV-2 residues 143 to 155), which overlays a cluster of aromatic residues (Figs. 2 and 5B). Both loop regions are variable, although the gate residues that replace biliverdin display considerable sequence conservation (consensus: ^175^[F,L]q[L,M]^177^; fig. S7). The marked gain in thermal stability upon biliverdin binding (fig. S3K) is consistent with resistance to an antibody that requires major conformational remodeling of the NTD for binding (movie S1).

## DISCUSSION

Our data show that the NTD of SARS-CoV-2 harbors dominant epitope(s), responsible for a considerable fraction of spike antigenicity (Figs. 3 and 4). It is well established that viruses use extensive glycosylation of their envelopes to shield antibody epitopes from recognition by humoral immunity (*24*–*26*). Here, we propose a novel mode of immune evasion via allostery, regulated through recruitment of a metabolite. In contrast to glycosylation, co-opting a metabolite may allow conditional unmasking, for example, under acidic conditions within the endosomal compartment. Notably, a recent study proposed pH-dependent conformational masking of the epitopes on SARS-CoV-2 RBD (*27*).

Since the beginning of the COVID-19 pandemic, considerable effort was directed to monitoring the genetic changes within the virus. The biliverdin-binding cleft has remained intact in the dominant SARS-CoV-2 variants (such as B.1.1.7, B.1.351, and B.1.1.28). However, while this manuscript was under review, R190S was reported in the emerging P.1 strain. It is currently unclear how widespread this mutation is, and it will be important to continue monitoring adaptation of the bat sarbecovirus to human host. Biliverdin levels in plasma of healthy individuals (0.9 to 6.9 μM) and more so under pathological conditions (>50 μM) (*28*) greatly exceed the *K*_d_ of its interaction with the spike (~10 nM) and are therefore sufficient to impinge on SARS-CoV-2 antigenic properties and neutralization. Although SARS-CoV-2 spike bound bilirubin with lower affinity (fig. S3), this final product of heme catabolism accumulates at higher levels in vivo (*28*). Elevated bilirubin levels correlate with the symptoms and mortality among patients with COVID-19 (*29*–*32*). Therefore, the tetrapyrroles may share a role in SARS-CoV-2 immune evasion. It is important to note that the binding of biliverdin and bilirubin to the spike is characterized by fast association and dissociation kinetics (fig. S3). The affinity of a typical antibody (10 to 200 pM) greatly exceeds that of the biliverdin-spike interaction (*33*). Accordingly, the metabolite is able to suppress antibody binding (Figs. 3 and 4), because it can reach considerably higher molar concentrations. In addition, tetrapyrrole levels are likely to vary between anatomical locations and during the course of natural infection, explaining the emergence of the biliverdin-sensitive antibody fraction.

Severe COVID-19 symptoms and death are associated with neutrophil infiltration in pulmonary capillaries and alveolar space (*34*). Nasopharyngeal swabs of patients with COVID-19 are enriched in neutrophil myeloperoxidase (*35*), a highly abundant heme-containing protein responsible for coloration of mucus (*36*). Incidentally, host mechanisms to minimize inflammation in viral infections include the activation of heme oxygenase 1 (*37*). Alongside extensive vascular damage, these symptoms provide rich source of heme catabolites, which may contribute to the inability to control the infection in severe cases. Although more work is required to validate this model, our results suggest that biliverdin binding may impair the sensitivity of SARS-CoV-2 immunoassays. Furthermore, it would be of interest to evaluate spike constructs deficient for the interaction with tetrapyrroles as vaccine candidates. Our results demonstrate a remarkable structural plasticity of the NTD and highlight the importance of this domain for antibody immunity against SARS-CoV-2.

## MATERIALS AND METHODS

### Protein expression and purification

DNA fragments encoding SARS-CoV-2 S1 (Uniprot ID: P0DTC2; residues 1 to 530), NTD (1 to 310), RBD (319 to 541), SARS-CoV-1 S1 (Uniprot ID: P59594; residues 1 to 518), HCoV NL63 (Uniprot ID: Q6Q1S2; residues 1 to 618), and HCoV OC43 (isolate LRTI_238, NCBI accession code KX344031; residues 1 to 619) were codon-optimized for expression in human cells and cloned control of the cytomegalovirus promoter for production of the recombinant proteins carrying a C-terminal extension containing human rhinovirus 14 3C protease recognition site followed by a TwinStrep tag. The signal peptide from immunoglobulin kappa gene product (METDTLLLWVLLLWVPGSTGD) was used to direct secretion of the RBD construct. The vector for production of the His_6_-tagged stabilized trimeric SARS-CoV-2 has been described (*18*). Expression constructs encoding heavy and light chains of P008_056 Fab were made by inserting the respective coding sequences into pHLsec (*38*), including a sequence encoding a hexa-histidine (His_6_) tag on the heavy chain fragment C terminus.

With exception of trimeric-stabilized SARS-CoV-2 spike ectodomain, the proteins were produced by transient transfection of Expi293 (Thermo Fisher Scientific) cells with endotoxin-free preparations of the corresponding DNA constructs using ExpiFectamine293 (Thermo Fisher Scientific). The cells were maintained in shake flasks in FreeStyle293 (Thermo Fisher Scientific) medium at 37°C in humidified 5% CO_2_ atmosphere. To produce SARS-CoV-2 S1 NTD fragment for crystallography, cell culture medium was supplemented with 5 μM kifunensine (Sigma-Aldrich) to suppress complex glycosylation (*39*). Conditioned medium containing recombinant product was harvested twice, 4 and 8 days after transfection, or once, for production of the NTD and P008_056 Fab, 5 days after transfection. For production of the trimeric SARS-CoV-2 spike ectodomain, Expi293 transfected with the pcDNA3-based expression construct (*18*) were selected with geneticin (250 μg/ml). Stably transfected cells, grown to a density of 3.5 million per milliliter at 37°C, were shifted to 32°C for 3 days before harvesting conditioned medium to enhance secretion of the viral glycoprotein (*40*).

TwinStrep-tagged proteins were captured on Strep-Tactin XT (IBA Lifesciences) affinity resin. Following extensive washes in TBSE [150 mM NaCl, 1 mM EDTA, and 25 mM tris-HCl (pH 8.0)], the proteins were eluted in 1× BXT buffer (IBA Lifesciences). His_6_-tagged proteins were captured on HisTrap Excel (Sigma-Aldrich) resin and eluted with 300 mM imidazole in phosphate-buffered saline (PBS). For the use in crystallography, SARS-CoV-2 S1 NTD was digested with Endo Hf (New England Biolabs) and rhinoviral 3C protease to trim glycans and to remove the C-terminal twin Strep tag; Endo Hf was depleted by absorption to amylose resin (New England Biolabs). The proteins were further purified by size exclusion chromatography through a Superdex 200 16/600 column (GE Healthcare) in HBSE [150 mM NaCl, 1 mM EDTA, and 20 mM Hepes-NaOH (pH 8.0)] and concentrated by ultrafiltration using a Vivaspin-20 with 10-kDa cutoff (Sartorius). To deplete biliverdin from SARS-CoV-2 S1, recombinant protein eluted from Strep-Tactin XT resin was supplemented with 0.5 M sodium acetate (pH 5.2) and subjected to size exclusion chromatography through a Superdex 200 16/600 column in 200 mM sodium acetate (pH 5.2); fractions containing S1 were pooled and dialyzed overnight against HBSE buffer. The light absorbance spectra of recombinant proteins were acquired using Jasco V-550 ultraviolet-visible spectrophotometer.

### Mass spectrometry

Recombinant SARS-CoV-2 S1 (3.5 mg/ml, 500 μl), denatured by addition of 1% (w/v) sodium dodecyl sulfate, was extracted with 500 μl of *n*-butanol. Organic phase containing green pigment was allowed to evaporate under vacuum, and dry residue was redissolved in 20 μl of water. Liquid chromatography–tandem MS (LC-MS/MS) was performed as described previously (*41*). LC-MS/MS analysis was conducted using a Dionex UltiMate LC system (Thermo Fisher Scientific) with a ZIC-pHILIC column (150 mm by 4.6 mm, 5-μm particle; Merck Sequant). A 15-min elution gradient of 80% solvent A [20 mM ammonium carbonate in Optima high-performance LC (HPLC) grade water; Sigma-Aldrich] to 20% solvent B (acetonitrile Optima HPLC grade, Sigma-Aldrich) was used, followed by a 5-min wash of 95:5 solvent A/solvent B and 5-min re-equilibration. Other parameters were as follows: flow rate, 300 μl/min; column temperature, 25°C; injection volume, 10 μl; and autosampler temperature, 4°C. Metabolites were detected across a mass range of 70 to 1050 mass/charge ratio (*m*/*z*) using a Q Exactive Orbitrap instrument (Thermo Fisher Scientific) with heated electrospray ionization and polarity switching mode at a resolution of 70,000 (at 200 *m*/*z*). MS parameters were as follows: spray voltage of 3.5 kV for positive mode and 3.2 kV for negative mode; probe temperature, 320°C; sheath gas, 30 arbitrary units; and auxiliary gas, 5 arbitrary units. Parallel reaction monitoring was used at a resolution of 17,500 to confirm the identification of biliverdin; collision energy was set at 30 in high-energy collisional dissociation mode. Data were recorded using Xcalibur 3.0.63 software and analyzed using Free Style 1.6 and Tracefinder 4.1 software (Thermo Fisher Scientific) according to the manufacturer’s workflows.

### Surface plasmon resonance

Experiments were performed on a Biacore S200 (Cytiva); S1 protein, diluted to 50 μg/ml in 10 mM sodium acetate (pH 5.0), was immobilized on a CM5 sensor chip (Cytiva product code BR100530) using amine coupling chemistry. Immobilization levels were typically 4000 response units. Analyte binding was studied in running buffer comprising 150 mM NaCl, 50 mM Hepes-NaOH (pH 8.0) or 50 mM bis-tris-HCl (pH 5.0), 0.05% Tween 20, and 1% dimethyl sulfoxide (DMSO). Biliverdin, bilirubin, heme, and protoporphyrin were obtained from Sigma-Aldrich (product codes 3089, 14370, 51280, and P8293, respectively). Generally, analyte stock solutions were prepared in DMSO before dilution in running buffer, maintaining the final DMSO concentration of 1%. The final analyte concentration was verified by spectrophotometry using the following molar extinction coefficients: biliverdin, 39,900 (at a wavelength of 388 nm); bilirubin, 53,846 (460 nm); heme, 58,440 (385 nm); and protoporphyrin IX, 107,000 (407 nm). Alternatively, biliverdin, which is highly soluble at pH > 7, was dissolved directly in running buffer to omit DMSO from the experiment. The presence of DMSO did not affect the measured *K*_d_ of the S1-biliverdin interaction (table S1). All experiments were conducted using a CM5-kinetics multicycle template at 25°C. Flow rate was 30 μl/min with a contact time of 180 s, followed by a dissociation time of 10 min; three start-ups were performed at the beginning of each experiment. Solvent correction was deemed unnecessary for the assays that contained DMSO. Biliverdin displayed very fast association precluding detailed kinetics analyses because of mass transfer effects. Data were analyzed using the 1:1 binding model in the Biacore S200 Evaluation software to estimate equilibrium binding constants (*K*_d_).

### Protein thermostability assay

Biliverdin-depleted SARS-CoV-2 NTD (corresponding to spike residues 1 to 310) was diluted to 1 mg/ml in 150 mM NaCl and 20 mM Hepes-NaOH (pH 8.0) and supplemented with biliverdin from a 5 mM stock prepared in 100 mM tris-HCl (pH 8.0) where appropriate. Melting curves were recorded using 20° to 95°C 1.5°C/min temperature ramps on a Promethius NT.48 instrument (Nanotemper). Melting points were determined from inflection points of fluorescence intensity ratios (350 and 330 nm) using first derivative analysis (fig. S3K).

### Cryo–electron microscopy

Four-microliter stabilized trimeric SARS-CoV-2 spike ectodomain (0.6 mg/ml final concentration in TBSE supplemented with 0.1% *n*-octyl glucoside) with biliverdin (25 μM ) or P008_056 Fab (0.2 mg/ml) was applied onto glow-discharged 200-mesh copper holey carbon R2/2 grids (Quantifoil) for 1 min, under 100% humidity at 20°C, before blotting for 3 to 4 s and plunge-freezing in liquid ethane using Vitrobot Mark IV (Thermo Fisher Scientific). The data were collected on Titan Krios microscopes operating at 300 keV (Thermo Fisher Scientific). Single particles of spike-biliverdin were imaged using a Falcon III direct electron detector (Thermo Fisher Scientific). A total of 15,962 movies were recorded with a calibrated pixel size of 1.09 Å and a total electron exposure of 33 e^−^/Å^2^ spread over 30 frames in single electron counting mode. The spike-Fab complex was imaged on a GIF Quantum K2 detector with a postcolumn energy filter (Gatan), selecting a 20-eV window, in single electron counting mode. A total of 17,010 movies were collected with a pixel size of 1.38 Å and total electron exposure of 51 e^−^/Å^2^ spread over 40 frames. Both datasets were acquired with a defocus range of −1.6 to −4 μm (table S2).

### Cryo-EM image processing and real-space refinement

Micrograph movies were aligned with dose weighting applied using MotionCor2 (*42*), and the contrast transfer function (CTF) parameters were estimated from the frame sums with Gctf (*43*). Images exhibiting ice contamination or poor CTF estimation were discarded at this stage, leaving 15,803 (biliverdin complex) and 16,619 (Fab complex) movies for further processing. An initial set of particles, autopicked with Laplacian-of-Gaussian function in Relion-3.1 (*44*), was used to generate 2D class averages that served as templates for picking both datasets with Gautomatch (www.mrc-lmb.cam.ac.uk/kzhang/). Particles (1,209,334 and 2,505,265 for spike-biliverdin and spike-Fab, respectively) were extracted from weighted frame sums, binned fourfold, and subjected to reference-free 2D classification in cryoSPARC-2 (*45*). A total of 371,422 spike-biliverdin and 709,127 spike-Fab particles belonging to well-defined classes containing trimeric spike were selected for further processing (figs. S4A and S11A). An initial 3D model was generated using ab initio procedure in cryoSPARC-2. Selected particles, re-extracted with twofold binning, were subjected to 3D classification into 11 (spike-biliverdin) and 16 (spike-Fab) classes in Relion-3.1. Particles belonging to selected 3D classes were re-extracted without binning and used in 3D reconstruction followed by CTF refinement (beam tilt and per-particle defocus) and Bayesian polishing in Relion-3.1. The final 3D reconstructions were done using nonuniform refinement procedure in cryoSPARC-2. Resolution is reported according to the gold-standard Fourier shell correlation using the 0.143 criterion (figs. S4, C and D, and S12, C and D, and table S2) (*46*, *47*). To aid in model building process, the maps were filtered and sharpened using deepEMhancer (*48*) or using density modification procedure in Phenix (*49*). An additional spike-Fab reconstruction was obtained using multibody refinement in Relion-3.1 accounting for two rigid bodies (one spanning the Fab moiety plus the associated NTD, and the second encompassing the rest of the structure) was used for illustration purposes (Fig. 4A).

For the 3RBD-down spike model, coordinates from protein databank entry 6ZGE (*18*) were docked into the cryo-EM map in Chimera (*50*). Residues 14 to 319 were replaced with the NTD crystal structure for each of the chains along with the associated biliverdin molecules. The model was manually adjusted in Coot (*51*) and refined using phenix.real_space_refine (version 1.19rc5-4047) (*52*). The model was then docked into the cryo-EM map of asymmetric 1RBD-up reconstruction and one RBD refitted to the extended position in Chimera; the model was manually adjusted in Coot and refined using phenix.real_space_refine. For the spike-Fab model, 3RBD-down spike model was fitted to the map using Chimera; the RBD of chain A was fitted in to the extended position, which is less well defined, and the NTD of chain C was extensively remodeled. The protein data bank was searched for similar structures using the sequence of the Fab chains; 6APC (*53*) and 6PHB (*54*) were selected as templates for the heavy and light chains of the Fab, respectively, and variable and constant subdomains were fitted individually using either phenix.dock_in_map (*55*) and/or fitted in Chimera. The Fab fragments were manually adjusted to match the sequence of P0008_056 antibody and fitted to the cryo-EM map. The constant domains of the Fab are less well resolved in the map, and minimal adjustments were made to these domains. The whole structure was subjected to automatic flexible fitting using Namdinator v2.13 (*56*) and then phenix.real_space_refine before another round of manual rebuilding in Coot and a final round of phenix.real_space_refine. Biliverdin (BLA) ligand geometry definition file was generated by Grade (Global Phasing), and model quality was assessed using Molprobity (*57*).

### Crystal structure of the NTD in complex with biliverdin

Protein construct (spanning SARS-CoV-2 S1 residues 1 to 310) at 10 mg/ml was supplemented with 90 μM biliverdin before mixing with crystallization mother liquor in a 1:1 ratio. Plate-like crystals grew to 80 to 120 μm in two dimensions and ~10 to 20 μm in the third dimension in conditions containing 24% polyethylene glycol, molecular weight 3350 (PEG-3350) (w/v) and 0.25 M NaSCN by hanging drop vapor diffusion over 1 to 2 weeks at 18°C. Crystals were cryoprotected by the addition of PEG-400 to a final concentration of 30% (v/v) to the drop solution and frozen by plunging in liquid nitrogen. X-ray diffraction data were collected at the PX1 beamline, Swiss Light Source using a wavelength of 1 Å, 100% transmission, a 40-μm beam, 0.1-s exposure, and 0.5° rotation per image. Data were indexed, scaled, and merged using XDS (*58*) and Aimless (*59*) via Xia2 (*60*). SARS-CoV-2 spike NTD (residues 14 to 290; Protein Data Bank ID 6ZGE) (*18*) was used as a model for molecular replacement and yielded a solution containing one NTD per asymmetric unit, with a log likelihood gain of 490 and translation function *z* score of 22.7, in space group C222_1_ using Phaser (*61*) within the Phenix package (*55*). The initial molecular replacement solution was subjected to morph model in Phenix before commencing with rounds of manual fitting in Coot (*51*) and refinement using phenix.refine (version 1.19rc4-4035) (*55*). First, the protein chain was fitted and extended where possible and refined, and then glycosylation moieties were added where visualized in the positive *Fo-Fc* density, followed by conceivable PEG and water molecules. The electron density around the disulfide bonds suggested that they were labile and, hence, were modeled as alternative conformations between oxidized and reduced where appropriate and the occupancy refined between these states. The stability of the disulfide bonds could have been affected by trace amounts of dithiothreitol introduced during the treatment of the protein with 3C protease and EndoH. The *R*_free_ and *R*_work_ were 21.5 and 18.5%, respectively, before a biliverdin molecule was fitted into the prominent positive difference density; rings A, B, and C are defined very well in the density, and ring D appears to be marginally less well defined. The final refinement included four Translation/Libration/Screw groups (residues 14 to 67, 68 to 202, 203 to 278, and 279 to 319) that had been segmented by the TLSMD server (*62*). Ligand geometry definitions were generated by Grade (Global Phasing). The final model consists of spike residues 14 to 319, 1 biliverdin molecule, 7 N-linked glycans (attached to asparagine residues at positions 17, 61, 122, 149, 165, 234, and 282), 10 PEG moieties, and 351 water molecules and has reasonable geometry and fit to the electron density (table S3 and fig. S5B). Model quality was assessed using Molprobity (*63*). The alignment of the sarbecoviral spike sequences with the NTD secondary structures (fig. S7) was formatted using ESPript3 server (http://espript.ibcp.fr/ESPript/ESPript/) (*64*).

### Human sera

Following written informed consent, serum samples from staff and patients of the Imperial College Healthcare NHS Trust and the Wellington Hospital diagnosed with SARS-CoV-2 infection were donated to the Communicable Diseases Research Tissue Bank (CDRTB) of the Section of Virology, Department of Infectious Disease, Imperial College London. The use of these sera was approved by the CDRTB Steering Committee in accordance with the responsibility delegated by the National Research Ethics Service (South Central Ethics Committee Oxford – C, NRES references 15/SC/0089 and 20/SC/0226). The median time from onset of symptoms [or positive reverse transcription polymerase chain reaction (RT-PCR) test in the case of asymptomatic infection] was 29 (0 to 94) days. In addition, serum or plasma samples were obtained from the University College London Hospitals (UCLH) COVID-19 patients testing positive for SARS-CoV-2 infection by RT–quantitative PCR and sampled between March 2020 and April 2020 (*23*). The sera were collected a median of 21 (9 to 31) days after onset of symptoms. Patient sera were from residual samples before discarding, in accordance with Royal College Pathologists guidelines and the UCLH Clinical Governance for assay development and approved by HRA (IRAS reference 284088). All serum or plasma samples were heat-treated at 56°C for 30 min before testing by flow cytometry.

### IgG capture assay

WT (depleted of biliverdin by chromatography under acidic conditions) and mutant SARS-CoV-2 S1 proteins (4.1 mg/ml; 100 μl) were conjugated to HRP using the Lynx Rapid HRP Conjugation Kit (Bio-Rad). Following quenching and dilution in conjugate stabilizer (Clintech, Guildford, UK; product code no. MI20080), half of each conjugate was supplemented with 10 μM biliverdin. Nunc 96-well, U8 MaxiSorp plates (Fisher Scientific) were coated overnight at 4°C with AffiniPure rabbit anti-human IgG antibody (Stratech, product code no. 309-005-008) diluted to 5 μg/ml in coating buffer (Clintech, product code no. 643005). Following a 3-hour incubation at 37°C and a 1-hour incubation at room temperature, the wells were washed with washing buffer (Clintech, product code no. 20024) and incubated for 4 hours in blocking solution (Clintech, product code no. MI20011). The wells were air-dried and stored desiccated at 4°C until use. For ELISA, 100-μl serum samples, each diluted 1:100 in diluent buffer (Clintech, product code no. 2040), were added to the coated wells and incubated stationary at 37°C for 1 hour. To detect S1-specific IgGs, the wells were washed with washing buffer (Clintech) and aspirated to dryness, following which 100 μl of S1-HRP fusion conjugate diluted in conjugate diluent (Clintech, product code no. 100171) to a previously defined optimum concentration (1:1500) was added and incubated for 1 hour at 37°C. The wells were then washed as before and developed for 30 min at 37°C using tetramethylbenzidine substrate (Clintech, product code no. 2030b) and quenched by the addition of 50 μl of stop solution (Clintech, product code no. 20031). The resulting optical densities (ODs) were acquired using a SpectraMax M2 reader (Molecular Devices).

The IgG capture ELISA data were modeled with a Bayesian linear model using the gamma likelihood function: Gamma(μ, Scale). The linear model took the form of log(μ) = intercept[Sample] + offset[Protein], where the intercept[Sample] term allows varying intercepts across samples [to account for the repeated measurements of each serum sample across conditions, assumed to be distributed as Gamma(μ_intercept_, scale_intercept_)] and the offset[Protein] term accounts for variation attributable to different protein coatings. Pairwise contrasts were drawn from the posterior distribution to construct credible intervals for the difference in OD values between different protein coatings. Priors: offset[Protein] ∼ Normal(−3,0.2), scale ∼ Exponential(5), μ_intercept_ ∼ Normal(1,0.2), scale_intercept_ ∼ Exponential(5). Monte Carlo settings: 10,000 iterations, four chains, adapt_delta = 0.95, and sampler = NUTS.

### Flow cytometry

Serum antibody binding to full-length SARS-CoV-2 S expressed on HEK293T cells was performed using a recently described method (*23*). Briefly, HEK293T cells were transfected with an expression vector (pcDNA3) carrying codon-optimized genes encoding either the WT SARS-CoV-2 S (UniProt ID: P0DTC2) or N121Q SARS-CoV-2 S using GeneJuice (EMD Millipore). Two days after transfection, cells were trypsinized and transferred into V-bottom 96-well plates (20,000 cells per well). Cells were incubated with sera (diluted 1:50 in PBS) for 30 min, with or without addition of 10 μM biliverdin throughout the staining period. They were then washed with fluorescence-activated cell sorting (FACS) buffer [PBS, 5% bovine serum albumin (BSA), and 0.05% sodium azide] and stained with fluorescein isothiocyanate anti-IgG (clone HP6017, BioLegend), allophycocyanin anti-IgM (clone MHM-88, BioLegend), and phycoerythrin anti-IgA (clone IS11-8E10, Miltenyi Biotech) for 30 min (all antibodies diluted 1:200 in FACS buffer). Samples were run on a Ze5 analyzer (Bio-Rad) running Bio-Rad Everest software v2.4 and analyzed using FlowJo v10 (Tree Star Inc.) analysis software. To calculate the effect of biliverdin, the mean fluorescence intensity (MFI) of positively stained cells only was used in each condition. The MFI data was modeled with a Bayesian linear model using the gamma likelihood function: Gamma(μ, Scale). The linear model took the form of log(μ) = intercept[Sample] + offset[Protein], where the intercept[Sample] term allows varying intercepts across samples [to account for the repeated measurements of each serum sample across conditions, assumed to be distributed as Normal(μ_intercept_,σ_intercept_)], and the offset[Protein] term accounts for variation attributable to different protein coatings. Pairwise contrasts were drawn from the posterior distribution to construct credible intervals for the difference in MFI values between samples ±biliverdin. Priors: offset[Protein] ∼ Normal(0,400), scale ∼ Exponential(0.1), μ_intercept_ ∼ Normal(2500,200), σ_intercept_ ∼ Exponential(0.01). Monte Carlo settings: 10,000 iterations, four chains, adapt_delta = 0.95, and sampler = NUTS.

The same procedure was performed to assess monoclonal IgG binding to cell surface SARS-CoV-2 spike with the following alterations: Transfection was performed with PEI-Max (1 mg/ml; Polysciences), FACS wash buffer (FWB) containing PBS supplemented with 1% BSA. Monoclonal IgGs were serially diluted 10-fold from 50 μg/ml before mixing with transfected cells. Antibody binding was detected with APC anti-IgG (BioLegend) diluted 1:200 in FWB buffer. Samples were run on a NovoCyte 96-well plate flow cytometer and analyzed using FlowJo v10 (Tree Star) analysis software. Three buffer only samples and secondary antibody alone conditions were used to define the spike-positive gate.

### Monoclonal human antibodies

The following IgGs—such as COVA1-26, COVA1-23, COVA2-38, COVA2-17, COVA1-20, COVA2-26, COVA1-22, COVA3-07, COVA2-03, COVA1-18,COVA1-12, COVA1-16, COVA2-01, COVA2-02, COVA2-04, COVA2-07, COVA2-11, COVA2-15, COVA2-29, COVA2-39, COVA2-44, COVA2-46, COVA2-10, COVA2-25, and COVA2-30—have been reported (*11*). Cloning and characterization of human IgGs P008_056, P003_027, P008_039, P008_051, P008_052, P003_014, P008_057, P008_100, P008_081, P008_017, P008_087, P054_021, P008_007, P003_055, and P008_108 are described elsewhere (*14*).

### ELISA with monoclonal IgGs

The assays were performed in a similar manner to the previously described protocol for serum samples (*23*, *65*, *66*). Briefly, high-binding ELISA plates (Corning, product code 3690) were coated with SARS-CoV2 WT S1 antigen (3 μg/ml; 25 μl per well) (purified with or without acid treatment) or N121Q S1 in PBS, either overnight at 4°C or for 2 hours at 37°C. Wells were washed with PBS supplemented with 0.05% Tween 20 (PBS-T) and blocked with 100 μl of 2% casein in PBS for 1 hour at room temperature. The wells were emptied, and 25 μl of 2% (w/v) casein in PBS was added per well. This solution was supplemented with biliverdin at 10 μM where indicated. Serial dilutions of IgGs were prepared in separate 96-well plate (Greiner Bio-One) in 2% casein, and then 25 μl of each serial dilution was added to the ELISA assay plates and incubated for 2 hours at room temperature. Wells were washed with PBS-T. Secondary antibody was added and incubated for 1 hour at room temperature. IgG binding was detected using goat-anti–human-Fc conjugated to alkaline phosphatase (1:1000; the Jackson laboratory, product code 109-055-098). Wells were washed with PBS-T, and alkaline phosphatase substrate (Sigma-Aldrich) was added and read at 405 nm. Area under the curve values were calculated using GraphPad Prism.

### Pseudotype infectivity assay

Simian immunodeficiency (SIV) particles were used to assess effects of H207A, R190K, and N121Q mutations on the function of SARS-CoV-2 spike. HEK293T cells, seeded 1 day earlier in 10-cm dishes in complete Dulbecco’s modified Eagle’s medium [DMEM; supplemented with 10% (v/v) fetal bovine serum (FBS)], were cotransfected with 17 μg of SIVMAC239–green fluorescent protein (GFP), an *env*- and *nef*-defective provirus construct expressing a GFP reporter (*67*), and 4 μg of pcDNA–SARS-CoV-2–del19, encoding SARS-CoV-2 spike with or without mutations in the biliverdin binding pocket. To improve pseudotyping efficiency, the constructs encoded a truncation of the spike C-terminal 19 amino acid residues (*68*). Viral pseudotypes were harvested 48 hours after transfection, clarified by low-speed centrifugation at 300*g* for 5 min, and filtered through a 0.45-μm filter. The stocks of viruses pseudotyped with the spike variants were diluted to an equal reverse transcriptase activity (*69*). Six fivefold serially diluted virus stocks were inoculated in quadruplicate in 96-well plates onto Huh7 and Vero cells, modified to overexpress ACE2 from a lentiviral vector, and seeded 1 day before infection in 96-well plates. At 48 hours after infection, fluorescent cells were counted using the Ensight plate reader (PerkinElmer). Infectivity was calculated from values falling into a linear dilution range by dividing the number of infected cells in a well for the amount of reverse transcriptase activity associated to the virus inoculum, expressed in mU.

### Pseudotype neutralization assay

HIV-1 particles pseudotyped with SARS-CoV-2 spike were produced in a T75 flask and seeded the day before with 3 million HEK293T/17 cells (American Type Culture Collection; catalog code CRL-11268) in 10 ml of complete DMEM supplemented with 10% (v/v) FBS, penicillin (100 IU/ml), and streptomycin (100 μg/ml). Cells were transfected using 60 μg of PEI-Max (Polysciences) with a mix of three plasmids: 9.1 μg of HIV-1 luciferase reporter vector (*65*), 9.1 μg of HIV-1 p8.91 packaging construct (*70*), and 1.4 μg of WT SARS-CoV-2 spike expression vector (*23*) or its N121Q mutant version. Supernatants containing pseudotyped virions were harvested 48 hours after transfection, filtered through a 0.45-μm filter, and stored at −80°C. Neutralization assays were conducted by serial dilution of monoclonal IgGs at the indicated concentrations in DMEM [10% (v/v) FBS and 1% penicillin-streptomycin] and incubated with pseudotyped virus for 1 hour at 37°C in 96-well plates. HeLa cells stably expressing ACE-2 (provided by J.E. Voss, Scripps Institute) (*71*) were then added to the assay (10,000 cells per 100 μl per well). After 48 to 72 hours, luminescence was assessed as a proxy of infection by lysing cells with the Bright-Glo Luciferase Kit (Promega) using a GloMax plate reader (Promega). Measurements were performed in duplicate and used to calculate 50% inhibitory concentrations in GraphPad Prism software.

### SARS-CoV-2 infectivity assay

SARS-CoV-2 strain BetaCoV/England/02/2020 (obtained from Public Health England) was propagated and quantified as described previously (*72*). VeroE6 cells were grown in DMEM (supplemented with 10% FBS) at 37°C and 5% CO_2_ in 96-well imaging plates (Greiner no. 655090). SARS-CoV-2 (0.5 plaque-forming units per cell) was added to DMEM with 2 or 10% (v/v) FBS with or without 100 μM biliverdin and added to the cells. At 22 hours after infection, cells were fixed, permeabilized, and stained for SARS-CoV-2 N protein using Alexa 488–labeled CR3009 antibody (*73*) and 4′,6-diamidino-2-phenylindole (DAPI). The plate was imaged using the high-content screening Opera Phenix microscope (PerkinElmer) with a 5× lens. Harmony software (PerkinElmer) was used to delineate the whole well area and to determine the total intensities of the Alexa 488 (SARS-CoV-2 N protein) and DAPI (which stains DNA) signals per said whole-well area during image acquisition. Background-subtracted Alexa 488 intensities were normalized to the untreated control condition.

### SARS-CoV-2 neutralization assay

Vero E6 (*Cercopithecus aethiops*–derived epithelial kidney cells, provided by W. Barclay, Imperial College London) cells were grown in DMEM supplemented with GlutaMAX (Thermo Fisher Scientific), 10% (v/v) FBS, and gentamicin (20 μg/ml) and incubated at 37°C in 5% CO_2_ atmosphere. SARS-CoV-2 Strain England 2 (England 02/2020/407073) was obtained from Public Health England. The virus was propagated by infecting Vero E6 cells in T75 flasks (60 to 70% confluent) at a multiplicity of infection 0.005 in 3 ml of DMEM supplemented with GlutaMAX and 10% (v/v) FBS. Cells were incubated for 1 hour at 37°C before adding 15 ml of the same medium. Supernatant was harvested 72 hours after infection following visible cytopathic effect and filtered through a 0.22-μm filter, aliquoted, and stored at −80°C. The infectious virus titer was determined by plaque assay in Vero E6 cells.

Neutralization assays were performed as previously described (*65*). Cells were seeded at a concentration of 20,000 cells per 100 μl per well in 96-well plates and allowed to adhere overnight. Serial dilutions of monoclonal antibodies were prepared with DMEM media [supplemented with 2% FBS, penicillin (100 IU/ml), and streptomycin (100 μg/ml); Thermo Fisher Scientific) and incubated with SARS-CoV-2 for 1 hour at 37°C. Biliverdin was added to the virus at a final concentration of 25 μM before addition to the antibody. The media was removed from the preplated Vero-E6 cells, and the serum-virus mixtures were added to the Vero E6 cells and incubated at 37°C for 24 hours. These virus/serum mixtures were aspirated, and cells were fixed with 150 μl of 4% formaldehyde at room temperature for 30 min and then topped up to 300 μl with PBS. The cells were washed once with PBS and permeabilized with 0.1% Triton X-100 in PBS at room temperature for 15 min. The cells were washed twice with PBS and blocked using 3% milk in PBS at room temperature for 15 min. The blocking solution was removed cells were incubated with SARS-CoV-2 nuclear protein–specific murinized CR3009 antibody (2 μg/ml) in PBS supplemented with 1% milk at room temperature for 45 min. The cells were washed twice with PBS, and horse anti-mouse-IgG–conjugated to HRP was added (1:2000 in 1% milk in PBS; Cell Signaling Technology, product code S7076) at room temperature for 45 min. The cells were washed twice with PBS, developed using 3,3′,5,5′-tetramethylbenzidine substrate for 30 min, and quenched using 2 M sulfuric acid before reading at 450 nm. Measurements were performed in duplicate.

## SUPPLEMENTARY MATERIALS

Supplementary material for this article is available at http://advances.sciencemag.org/cgi/content/full/sciadv.abg7607/DC1

## Appendix

View/request a protocol for this paper from *Bio-protocol*.
